# Can We “Hedge” against the Development of Antiviral Resistance among Pandemic Influenza Viruses?

**DOI:** 10.1371/journal.pmed.1000103

**Published:** 2009-06-30

**Authors:** David K. Shay, Benjamin J. Ridenhour

**Affiliations:** Influenza Division, United States Centers for Disease Control and Prevention, Atlanta, Georgia, United States of America

## Abstract

David K. Shay and Benjamin Ridenhour discuss a modeling study predicting that stockpiling a secondary antiviral for use early in a flu pandemic can forestall resistance to the primary stockpiled drug.

**Linked Research Article**

This Perspective discusses the following study published in *PLoS Medicine*:

Wu JT, Leung GM, Lipsitch M, Cooper BS, Riley S (2009) Hedging against Antiviral Resistance during the Next Influenza Pandemic Using Small Stockpiles of an Alternative Chemotherapy. PLoS Med 6(5): e1000085. doi:10.1371/journal.pmed.1000085

Mathematically simulating an influenza pandemic, Joseph Wu and colleagues predict that using a secondary antiviral drug early in local epidemics would reduce global emergence of resistance to the primary stockpiled drug.

In the May 2009 issue of *PLoS Medicine*, Wu and colleagues presented the results of a series of mathematical models that examine how the use of influenza antiviral drugs might influence the development of antiviral resistance [1]. Their analyses suggest that a small supplemental stockpile of an alternative antiviral, in addition to a primary stockpiled antiviral (oseltamivir for every country that has stockpiled antivirals to date), could reduce levels of resistance to the primary drug during the early phases of an influenza pandemic. The recent emergence and spread of a novel influenza A(H1N1) virus in North America makes these results timely and potentially important to the world's response to this virus [2]. Can we use the study by Wu et al. to inform current public policy development to respond to this pandemic threat?

The authors used a standard compartmental model—in this case, one similar to a susceptible–exposed–infectious–recovered or SEIR model—to perform a series of stochastic simulations. In their model, infected individuals spent time first in a latent stage, then in a presymptomatic but infectious stage, and finally in either an asymptomatic stage or a symptomatic stage. At the beginning of a simulation, only susceptible influenza strains existed in the population; drug-resistant viral strains arose at a fixed probability and only in individuals receiving treatment. Using this framework, the authors considered three treatment scenarios. The ultimate objective was to determine which strategy resulted in the fewest pandemic influenza cases infected with an antiviral-resistant virus. These scenarios were: (1) monotherapy using oseltamivir, (2) early combination chemotherapy (ECC), and (3) sequential multidrug chemotherapy (SMC). The latter two scenarios were the “hedging strategies” considered in this study. For these two scenarios, a secondary antiviral stockpile sufficient to treat 1% of the population was established. In the ECC strategy, individuals receive both the primary and secondary antiviral until the stockpile of the secondary drug is depleted; thereafter, individuals received only the primary stockpile. The SMC strategy dictates that the secondary antiviral was used until depleted and thereafter the primary antiviral was used. Based on the currently available medications, this secondary antiviral would be either zanamivir (a neuraminidase inhibitor like oseltamivir, but one with a different resistance profile) or an M2 ion channel inhibitor (either amantadine or rimantadine). Using this model, the authors address the important question of how best to minimize antiviral resistance in circulating influenza strains. It should be noted the novel influenza H1N1 virus tested to date have all demonstrated susceptibility to both the neuraminidase inhibitors and resistance to the M2 ion channel inhibitors (http://www.cdc.gov/flu/weekly/).

Several features of influenza viruses are important in evaluating how well the authors' model can address their primary question. One of these is the extent to which drug resistance develops among influenza viruses and how it becomes widespread. It is unknown how resistance to M2 ion channel blockers or neuraminidase inhibitors rapidly became widespread among various strains of influenza A viruses. In particular, it is not clear whether the spread of antiviral-resistant viruses was due to a direct selective advantage of the mutations responsible for antiviral resistance or a “hitchhiker effect” in which drug resistance mutations were carried along with others that offered an advantageous immunological niche for a particular influenza strain. Their model assumes that use of antivirals increases the chance that resistant viruses will be isolated—and indeed a substantial correlation has often been demonstrated between widespread use of a particular antimicrobial agent and the prevalence of resistant organisms [3]—but the milieu in which influenza antiviral drug resistance develops is more complex. For example, from the licensure of the neuraminidase inhibitors oseltamivir and zanamivir in 1999 until recently, resistance to these agents had remained at a low level, even in the countries responsible for most of their use worldwide [4]. When neuraminidase inhibitor-resistant influenza viruses were detected, they were isolated from treated individuals and generally showed reduced fitness, as defined by their ability to transmit from individual to individual [5]. These seemingly well-established tenets had to be reevaluated rapidly with the emergence of seasonal influenza A(H1N1) viruses resistant to oseltamivir in the 2007–2008 season [6]. These viruses were clearly transmissible from person to person, and none of the 99 individuals infected with these viruses who were carefully evaluated had been exposed to oseltamivir prior to diagnosis [6]. Moreover, in some countries with high levels of oseltamivir resistance (e.g., Norway), oseltamivir was only rarely prescribed, while resistance was rare in Japan, where use of this drug was the most common [7].

These basic questions surrounding the epidemiology of antiviral resistance have important ramifications for the use of models in evaluating antiviral strategies. Given the complexities of the relationships between the use of antivirals and the prevalence of infections with resistant viruses at a country level, and our lack of understanding of why transmissible oseltamivir-resistant viruses suddenly emerged, including a parameter in the model to account for a potential reduction in the transmissibility of resistant viruses would have been useful. The lack of a term for a reduction in transmissibility may be a factor leading to the high attack rates of antiviral-resistant influenza produced by this model. Another effect of not considering that lower-fitness antiviral-resistant mutants may develop is that the model represents essentially a best-case scenario for hedging strategies (because the numbers of antiviral-resistant influenza cases against which to hedge may have been artificially increased). Finally, the assumption of a synergistic effect of two-drug treatment among individuals receiving ECC (i.e., the occurrence of resistance becomes less likely) critically depends upon the magnitude of this effect. ECC might represent the optimal strategy if the synergistic effect is as great as that suspected for a combination of oseltamivir and amantadine, based on in vitro results [8]. The magnitude of any synergy coefficient has no effect on the SMC or the monotherapy strategies. It is also worth discussing the authors' use of fixed rates of acquisition of antiviral resistance in their model. While they do consider a plausible range of fixed rates, there are at least two potential problems with their approach: (1) published estimates of these rates for oseltamivir and amantadine seem to be at the higher end of the investigated range (for which the model predicts the choice between hedging strategies may make little difference) and (2) the age of the individual being treated has an effect on the development of antiviral resistance, suggesting that an age-stratified compartment model would be more appropriate. Use of such a model may (or may not) produce different results; it would be informative to check.

The authors used an extensive database of air-travel data in this study, which allowed them to model the dissemination of infected individuals to a global network of 105 cities based on known travel patterns; in the modeled scenarios 28 of these cities employed large-scale antiviral campaigns (24 at random, with Hong Kong, London, Geneva, and New York always participating). The authors demonstrate that applying an effective hedging strategy at the source (Hong Kong in this case) has dramatic effects on the attack rates of resistant viruses in the other three major cities, and more globally as well. Of course, the interpretation of this finding is contingent upon the resemblance of this air-travel model to the current, complex reality of global travel patterns, not all of which can be captured using data on regularly scheduled commercial airline flights.

The interpretation of results from all influenza modeling studies depends on the reasonableness of the input assumptions used, in light of current epidemiologic characteristics of the virus and its human host and the relevance of the model outputs to plausible public health interventions. As the authors note, limitations of the current study included an assumption that the pandemic virus would be susceptible to both antiviral agents in the putative stockpile when it was detected at a major city, and that its spread would occur primarily to other major cities throughout the world through air-travel routes. Another limitation is the current lack of clinical safety data for antiviral drug combinations.

As of late May 2009, the latest and most pressing pandemic threat is a novel influenza A(H1N1) virus resistant to the M2 ion channel inhibitors that was first detected as a cause of community-level respiratory illness outbreaks in Mexico. This virus spread to the United States quickly but not solely or even primarily by the air-travel network and has caused outbreaks in rural and urban areas. For their work to be most useful, modeling groups should work closely with epidemiologists and public health officials to evaluate control strategies that appear feasible from epidemiologic, logistical, and regulatory perspectives. Collaborations between modelers and public health scientists are forming rapidly during the current response to outbreaks of novel H1N1 infections in the Northern Hemisphere. We hope such collaborations will be able to provide timely data to help inform policy decisions regarding management of novel H1N1 outbreaks in the Southern Hemisphere in the next few months.

## Abbreviations

ECC: early combination chemotherapy
SMC: sequential multidrug chemotherapy
